# Automatic and Precise Localization and Cortical Labeling of Subdural and Depth Intracranial Electrodes

**DOI:** 10.3389/fninf.2017.00010

**Published:** 2017-02-17

**Authors:** Chaoyi Qin, Zheng Tan, Yali Pan, Yanyan Li, Lin Wang, Liankun Ren, Wenjing Zhou, Liang Wang

**Affiliations:** ^1^Chinese Academy of Sciences Key Laboratory of Mental Health, Institute of PsychologyBeijing, China; ^2^Department of Psychology, University of Chinese Academy of SciencesBeijing, China; ^3^Beijing Key Laboratory of Neuromodulation, Beijing Institute of Functional Neurosurgery, Xuanwu Hospital, Capital Medical UniversityBeijing, China; ^4^Epilepsy Center of Yuquan Hospital, Tsinghua UniversityBeijing, China; ^5^Chinese Academy of Sciences Center for Excellence in Brain Science and Intelligence TechnologyShanghai, China

**Keywords:** intracranial EEG, SEEG, ECoG, electrode localization, intractable epilepsy

## Abstract

**Object:** Subdural or deep intracerebral electrodes are essential in order to precisely localize epileptic zone in patients with medically intractable epilepsy. Precise localization of the implanted electrodes is critical to clinical diagnosing and treatment as well as for scientific studies. In this study, we sought to automatically and precisely extract intracranial electrodes using pre-operative MRI and post-operative CT images.

**Method:** The subdural and depth intracranial electrodes were readily detected using clustering-based segmentation. Depth electrodes were tracked by fitting a quadratic curve to account for potential bending during the neurosurgery. The identified electrodes can be manipulated using a graphic interface and labeled to cortical areas in individual native space based on anatomical parcellation and displayed in the volume and surface space.

**Results**: The electrodes' localizations were validated with high precision. The electrode coordinates were normalized to a standard space. Moreover, the probabilistic value being to a specific area or a functional network was provided.

**Conclusions:** We developed an integrative toolbox to reconstruct and label the intracranial electrodes implanted in the patients with medically intractable epilepsy. This toolbox provided a convenient way to allow inter-subject comparisons and relation of intracranial EEG findings to the larger body of neuroimaging literature.

## Introduction

Intracranial electroencephalograph techniques, including electrocorticography (ECoG) and stereotactic electroencephalography (SEEG), have been widely used in clinics to assess the localization of seizure onset zone for patients with medically intractable epilepsy. In addition to the remarkable contribution to clinical diagnosis, this technology provides a window to investigate neural mechanisms of human cognitive functions (Engel et al., 2005; Gonzalez-Martinez et al., 2013; Wang et al., 2016).

For surgical planning and neurobiological studies, a crucial point is to precisely determine anatomical localizations of the implanted electrodes. There have been several useful approaches developed to accomplish this goal (Morris et al., 2004; Hunter et al., 2005; Kovalev et al., 2005; Sebastiano et al., 2006; Hermes et al., 2010; Dykstra et al., 2012; Yang et al., 2012; Princich et al., 2013; Arnulfo et al., 2015). For examples, early studies (Hunter et al., 2005; Sebastiano et al., 2006) tried to use single CT or X-ray photos to reconstruct the cortical surface and localize the electrode, which was simple and precise within modality, but it suffered from the poor electrode/skull and tissue contrast and was not able to deal with the brain shift problem after electrode implantation. One recent method proposed (Arnulfo et al., 2015) introduced an appropriate way to automatically segment the SEEG contacts with good reliability, but their method requires two high-resolution CT images (i.e., before and after electrode implantation), which might be unconventional for many hospitals. This constraint also applied to the previous studies using two MRI images (Kovalev et al., 2005). So far, few studies have developed different methods to localize ECoG electrodes in a manual or semi-automatic manner. However, these methods are accompanied by more or less deficiencies, such as relatively time consuming, unsatisfied accuracy, no precisely cortical information or only application to subdural electrodes.

In this study, we proposed an integrative solution to localize both SEEG and ECoG electrodes based on pre-operative T1 and post-operative CT. The identified electrodes can be marked to cortical labels and rendered to a brain surface in the native space and a standard space for group statistics. Notably, we also provided probabilistic values of each contact to be a brain area and functional network according to either anatomical or function atlas (e.g., Brodmann areas and fMRI-based functional networks). Those functions have been integrated into an open MATLAB toolbox, which can be freely downloaded. This study is aimed to provide a practical tool for the precise localization and representation of intracranial electrodes and to advance the progressing of clinical and cognitive studies.

## Methods and materials

### Subjects

Patients with medically intractable epilepsy underwent routine long-term video monitoring to assess the seizure onset zone. High-resolution structural T1 image was acquired before electrode implantation. After surgical operation, a CT image was acquired for roughly checking electrode locations. Patients with either SEEG or ECoG electrodes implanted were included in this study.

The SEEG depth electrodes usually contain 8–16 contacts (DIXI Medical, Lyon). Each contact is a platinum-iridium cylinder of 0.8 mm diameter, 2 mm long with 1.5 mm of inter-contact distance.

The ECoG subdural electrodes had 4 mm diameter (2.3 mm exposed), 1 cm inter-electrode distance, and were embedded in silastic (Adtech Medical, Racine, WI). Electrode selections and locations were determined merely by neurosurgeons based on clinical perspective. Informed consent was obtained from all subjects and procedures were approved by the ethical committee of Institute of Psychology, Chinese Academy of Sciences.

### Data acquisition

MRI scanning was performed at the either Tsinghua university or Xuanwu hospitals using a 3T scanner prior to electrode implantation. The T1-weighted images were acquired with final in-plane isotropic resolution of 1 mm (TR/TE/TI = 7.8/3.1/400 ms, matrix = 256 × 256, FOV = 256 × 256 mm, and 190 slices) in ~7 min.

CT scanning was always performed immediately after placement of electrodes and as part of the clinical protocol for the evaluation of possible complications such as hematoma, contusions or subdural effusions. CT images were acquired using 215 mm FOV, Matrix = 512 × 512, 0.8 mm slice thickness, 207 slices with in-plane isotropic reconstruction of 0.42 mm in ~3 min.

### Pre-processing

Both T1 and CT images of each patient was imported from the scanner as DICOM format and transformed to NITFI format (http://nifti.nimh.nih.gov). A basic reconstruction step was performed on pre-implant structural T1 image to get an individual cortex parcellation for each patient using Freesurfer (http://surfer.nmr.mgh.harvard.edu/). This reconstruction process would take several hours and can be largely accelerated using computing cluster. The outputs were carefully checked for each subject.

The cortical parcellation was based on gyral and sulcal structures (Fischl et al., 2004; Desikan et al., 2006). To achieve this automatic parcellation, the pial surfaces are inflated to obtain a sphere (Fischl et al., 1999a) and registered to a spherical atlas. This atlas used individual cortical folding patterns to match a group-based cortical geometry (Fischl et al., 1999b). Freesurfer's automatic surface extraction and parcellation procedures have been demonstrated to show good test-retest reliability across scanner manufacturers and across field strengths (Han et al., 2006; Reuter et al., 2012). Moreover, this step has been validated by measuring mean distance of error maps for cortical labels on the brain surface and revealed that the mismatch is minimal (Desikan et al., 2006; Klein and Tourville, 2012). We then co-registered individual CT to the T1 image in Freesurfer's conformed space using normalized mutual information function. The mutual information function has been approved to be effective by several studies in the co-registration of T1 and CT images (Studholme et al., 1995; Maes et al., 1997).

### Image display and surface render

One of basic functions was to simultaneously display a volume image (CT or T1) and the corresponding 3D brain surface (e.g., pial surface) and showed the cursor location in two spaces in real time. The used surface included the conventional pial surface and the brain-outline surface which was separately created and smoothed for better performance. The brain outlined surface was created using the matlab function isosurface to computes isosurface data (vertices and faces) from the brain volume generated by Freesurfer. The cortical parcellation from Freesurfer was also used for color-coding a brain region in a native volume.

The density map was calculated to represent a set of overlapped regions (e.g., activations in specific electrode sites). The density map used a gaussian kernel centered at the coordinate of each electrode with different Full width at half maximum (FWHM = 5 mm for ECoG and 2 mm for SEEG) which was approximately equal to electrode length (Tsuchiya et al., 2008). The density *D*_*i*_ of the electrode i is denote as

$$D_{i}=\frac{w_{i}e^{-(x_{i}^{2}\;+\;y_{i}^{2}\;+\;z_{i}^{2})}}{2c^{2}}$$

where $c=\frac{FWHM}{2\sqrt{2log2}}$. The *w*_*i*_ indicates the weight for the electrode i (e.g., equal to 1 at default to represent electrode density maps or power spectrum for brain activity maps). x_i_/y_i_/z_i_ are the coordinate of the electrode i in the standard space.

A full electrode-based display for SEEG can be completed by obliquely reslicing the original volume to show the full electrode in a coronal view. The peri-coronal plane was calculated as follow,

$$\begin{array}{l} V_{a}=V_{c}\times V_{r}\\ \;\theta =acos(V_{c}\cdot V_{r}) \end{array}$$

where *V*_*r*_ is the norm vector of the slicing plane defined by the most outside and inside brain contacts (i.e., two ends); *V*_*c*_ is the norm vector of a constructed plane (e.g., [0 0 1] for a coronal view); *V*_*a*_ is a norm vector representing the rotation axis from the center of the plot box.

### Localization of intracranial electrodes

The most difficulties involving in the segmentation of intracranial electrodes using post-operative CT image were the skull signals and the noises induced by the interpolation error during the coregistration step. To solve these problems, a skull stripping process was essential. Previous studies proposed some methods, such as a single threshold filter or subtraction between two same modality images with and without electrodes implanted (Morris et al., 2004; Kovalev et al., 2005; Sebastiano et al., 2006; Tao et al., 2009; Yang et al., 2012; Arnulfo et al., 2015). For the first case, a single value threshold filter was the most simple and applicable way to remove the majority of skull tissue in a CT image, but it was very rough and might leave a lot of scattered noises that significantly affected the segmentation of the real electrodes. In contrast, the subtraction of two sets of same modality image (one pre-surgical and one post-surgical) was a very efficient way to remove the skull and leave a clean image containing the real electrodes. However, two high quality CT images were usually unconventional in many hospitals' clinical procedures. Here we proposed two semi-auto segmentation methods using different skull stripping processes for SEEG and ECoG electrodes, respectively.

#### ECoG

For subdural electrode arrays with or without depth electrodes, a good skull stripping process was critical to the performance of an auto-segmentation method. Since ECoG contacts touched the cortical surface and they were also close to the inner skull boundary, it was difficult to implement a skull stripping without removing any contacts in a CT image. Here we proposed a skull stripping method utilizing two brain masks to extract the most brain tissue with implanted electrodes.

The basic skull stripping mask was taken from the freesurfer output (i.e., brain.mgz with the skull properly stripped) which denoted all the brain tissue separated from the T1 image. This image was threshold and binarized. Then the brain mask was slightly dilated by adding several layers of pixels to the boundaries of the mask volume to make another brain mask. Two masks were separately multiplied with the coregistered CT image and threshold to get two masked CT images. Automated connectivity based cluster segmentation was implemented for the two masked CT images separately. We assumed that the voxels belonging to clusters of different size in the two masked images should be skull voxels. Based on this assumption, the clusters containing those voxels were removed from further rectification. This step eliminated the most of non-electrode tissues and retained the most of valid clusters. The remaining clusters (i.e., electrode's contacts) were put into a threshold iteration process using inter-cluster distance as constraints. The initial cluster number was usually larger than the predefined number(default is the total contact number). Subsequently, the process automatically adjusted the threshold to a level higher than initial settings during the auto-segmentation procedure. Then size of each cluster and number of clusters can be reduced dramatically. Then an inter-cluster distance would be calculated. The clusters that have less than 2 near neighbors would be considered as irrelevant tissue and removed according to the spatial distribution of the electrode array. The cluster number was counted in each iteration and compared with the predefined number. Once the cluster number was very close or equal to the predefined number (±5%), the iteration was terminated and the centroid of the remaining clusters denoted the contact coordinates. As noted by other iEEG segmentation methods, a manual validation was still needed to remove any incorrect contact points and add lost points. This correction can be done more easily here by referring to the neighboring contacts' positions on the rendered surface and only took about several minutes.

After rectifying all the contacts' positions, the updated coordinates can be output. In order to decrease the influence of brain deformation caused by the electrode implantation (Hill et al., 1998), our approach had integrated a minimal energy projection method proposed recently (Dykstra et al., 2012) to project the electrodes to the brain surface.

#### SEEG

For stereotactic depth electrodes, we performed a different semi-automatic segmentation process. Those electrodes were usually connected with the skull in a CT volume. Though a high value threshold can separate the electrode clusters from the skull, there raised a risk in cutting off electrodes with relative low intensity that could be caused by different CT scan angles. Thus, the ECoG-based skull stripping process can not be applied to SEEG electrode segmentation.

**Figure 1:**
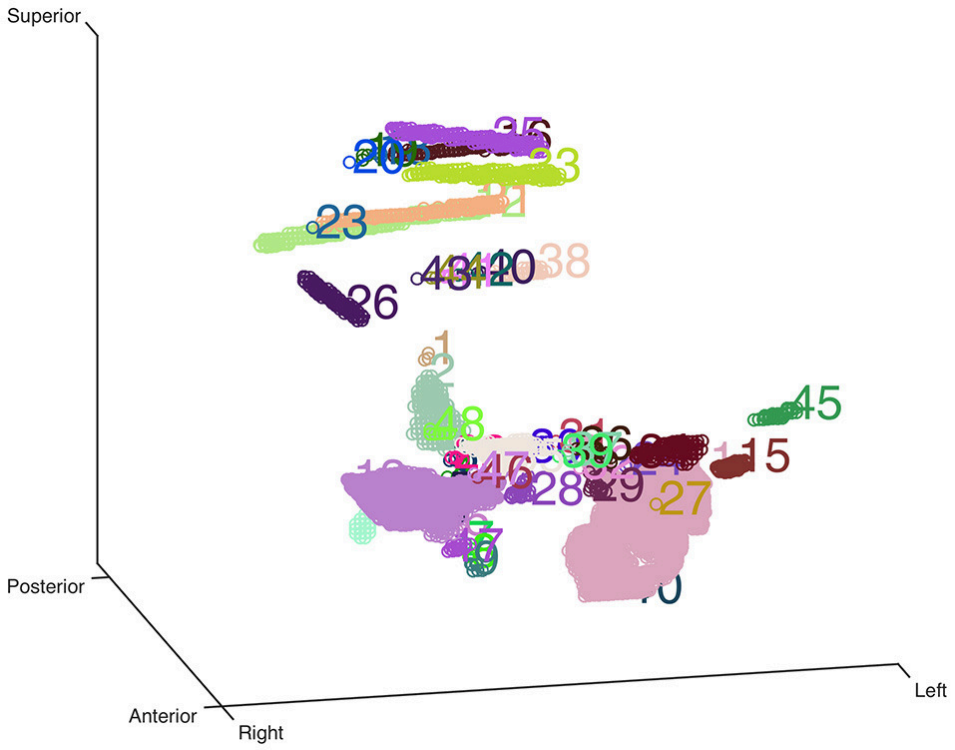
**Clustering-based automatic segmentation for electrode detection**. Color-coded clusters with unique number are displayed in a stereo space. The real electrode-related clusters can be detected by specifying the corresponding cluster number. Rotating and zooming functions can be used to facilitate this process.

**Figure 2:**
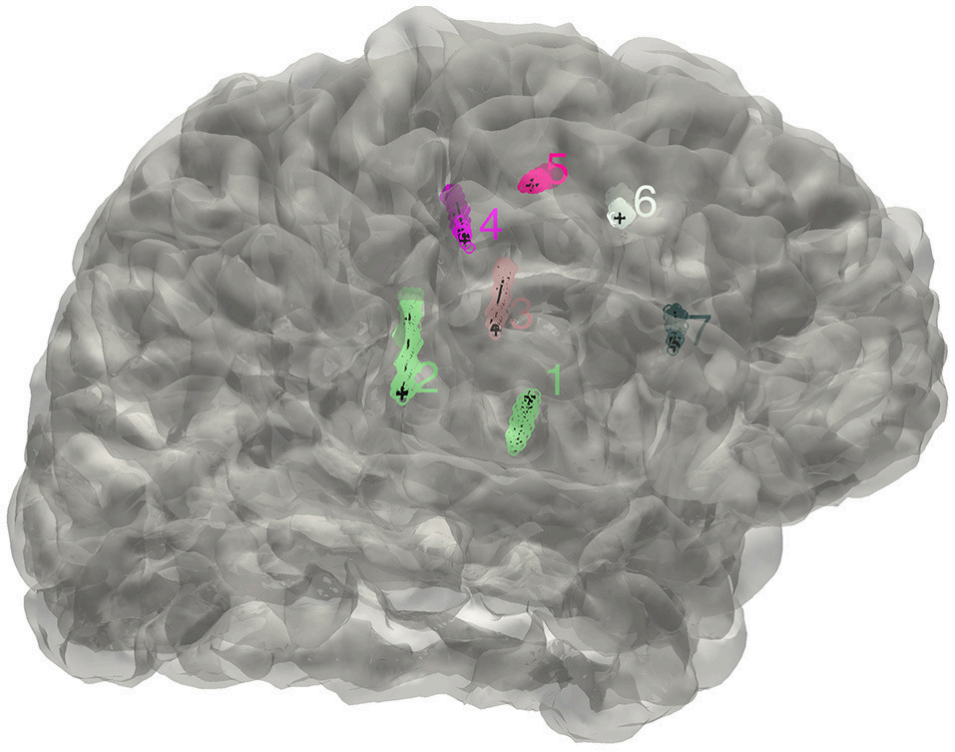
**The distribution of electrodes**. The clusters detected for the implanted electrodes are superimposed on the individual brain surface. The electrodes shown can be rearranged in a right order if needed.

Here we used an eroded brain mask to multiply with the CT volume (eroded 4 mm as default) to cut off the majority of the outer skull tissue to reveal the electrodes position. A single value threshold was applied to remove some irrelevant points. Then the electrode clusters were detected in the masked volume. As shown in Figure 1, electrode clusters together with some outer skull tissue were displayed. The figure can be rotated and zoomed for a better view. An instant threshold adjustment can be performed to separate near or cross electrode clusters and exclude disturbing clusters. Based on the differences in spatial distribution of electrode clusters and non-electrode clusters, it was easy to choose the clusters for real electrode by an interactive interface. After all electrode clusters have been chosen correctly, we can view the electrodes' relative positions on the rendered surface and edit the electrode orders if needed (Figure 2).

Once all the electrodes were rearranged in a correct order, all the contacts along each electrode were tracked out automatically. In this step, each electrode track was fitted to a curve. To estimate the electrode's trajectory as accuracy as possible, we tested different order for curves to fit the electrode cluster and concluded that a cubic curve was sufficient for almost all of the electrodes, except for very rare patients where a high order of curve was required. The fitted trajectory of each electrode was represented as a bunch of equally spaced dots. For each electrode trajectory, two farthest points were first defined to determine the starting point. As a common situation, an electrode stretched out in one direction and the most inside point was defined as the starting or deepest point of the electrode trajectory. A similar centroid estimation step (Arnulfo et al., 2015) was then applied to the starting point to get a more precise approximation of the optimized electrode contact. The same centroid processes were applied to other contacts along each electrode one by one. This step should follow two spacial constraints: (1) the fixed inter-contact center distance (i.e., 3.5 mm); (2) the axis deviations occurring within electrode trajectory under a predefined level. For those electrodes that could be cut off in the first threshold process at the removal of skull, the missing contacts can be automatically padded based on the contact number of each electrode.

### Anatomical localization and atlas-based label

The iEEG contact can be anatomically labeled with a cortical area based on Freesurfer's pacellation (Fischl et al., 2002) in native space. So far, many atlases have been created using functional and structural imaging data and can be downloaded freely (Mazziotta et al., 2001; Desikan et al., 2006; Yeo et al., 2011). Thus, we also sought to label all converted iEEG contacts in standard space based on those atlases.

Spherial ROI (radius = 3.5 mm for SEEG and 5 mm for ECoG) was created for each contact around the electrode coordinate in native space. The transformation parameters were calculated based on the transformation of individual T1 image to a MNI template and then applied to each spheral ROI. Thus, all electrodes can be pooled together in the standard space.

For both anatomical and functional localizations, the region label of each ROI was determined by comparing the probabilities of all tissues included in the ROI as implemented in our previous study (Wang et al., 2015). Briefly, to avoid assigning a ROI to a region label with low probability, we first calculated the percentage of voxels labeled as gray matter (anatomical localization) or all atlas-based areas (functional localization) within the ROIs (inclusion) and compare that to the percentage of voxels in this ROI being outside (exclusion). If the probability of inclusion exceeded that of exclusion, the ROI was determined to be in the gray matter; otherwise, it was marked to be in the white matter or unknown area. For each included ROI, the probability values were compared between brain areas involved and the ROI was labeled as the structural or functional area with maximal probability value.

### Validation of SEEG electrodes reconstruction

**Figure 3:**
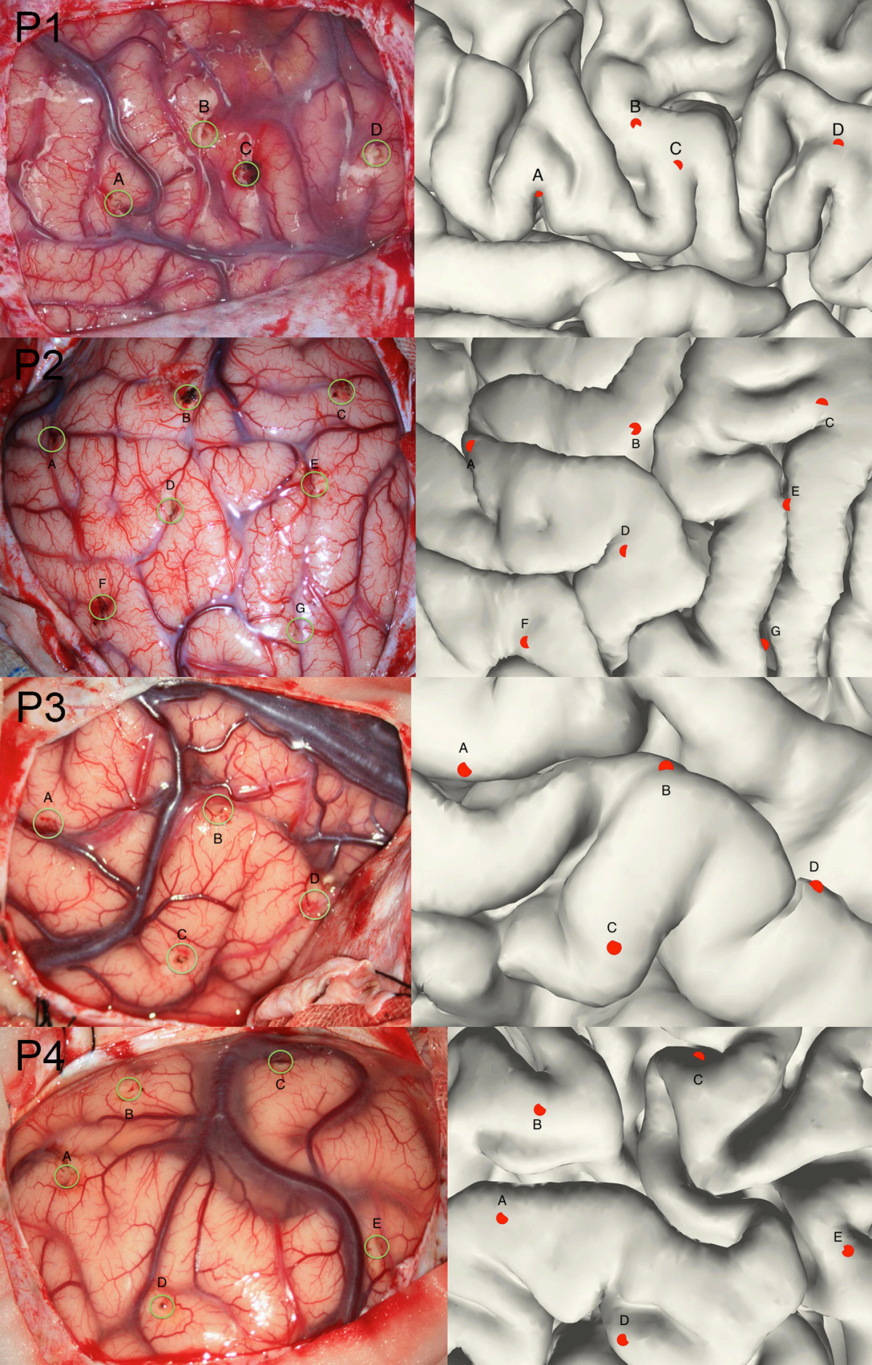
**Validation of the electrode reconstruction**. The points marked by A, B, C, D, E, F, and G are the outer positions of the depth electrodes included in the validation step for 4 patients. The left panel denotes the real electrodes' positions from an intraoperative picture and the right panel denotes the reconstructed electrodes' positions on the pial.

Though the SEEG contacts were directly detected from the coregistered MRI and CT images, it was important to validate the reconstruction results. The intraoperative picture can clearly show some electrode traces marked by the wounded patches on the brain tissue. These patches were used to compare with the reconstructed sites along the tissue. The position of the electrode track on the cortical surface was determined by the cross point between the reconstructed electrode trajectory and the brain surface (Figure 3). Notably, this approach cannot fully validate all the localization of SEEG electrodes only because the superficial positions can be photographed.

## Results

### Volume display and surface rendering

Real-time updating cursor coordinate, image intensity, and anatomical information were displayed in a concise panel. The parcellation map obtained from Freesurfer can be superimposed and color-coded on the origin image. A yoked window displaying the rendered individual surface was invisible by default and can be turned on and off at any time. The transparency of the surface can be adjusted for a clear representation of the cortical gyri.

**Figure 4:**
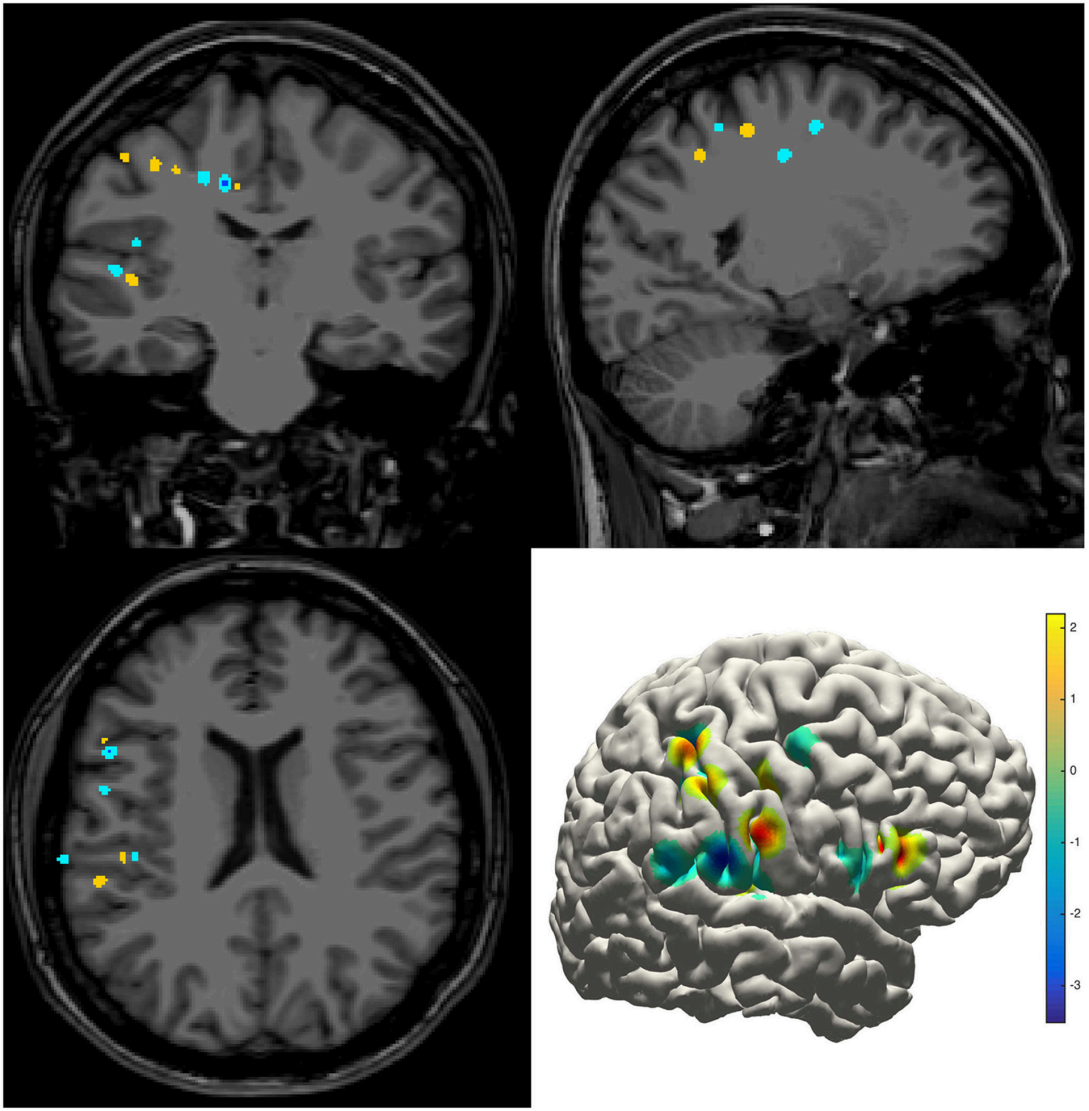
**Density map**. Volume and surface view of a density map (here for instance brain activity map shown in power spectrum). The warm-color voxels indicate the positive value and cool-color for the negative value. The volume image was displayed in a radiology view (i.e., left side = right hemisphere).

**Figure 5:**
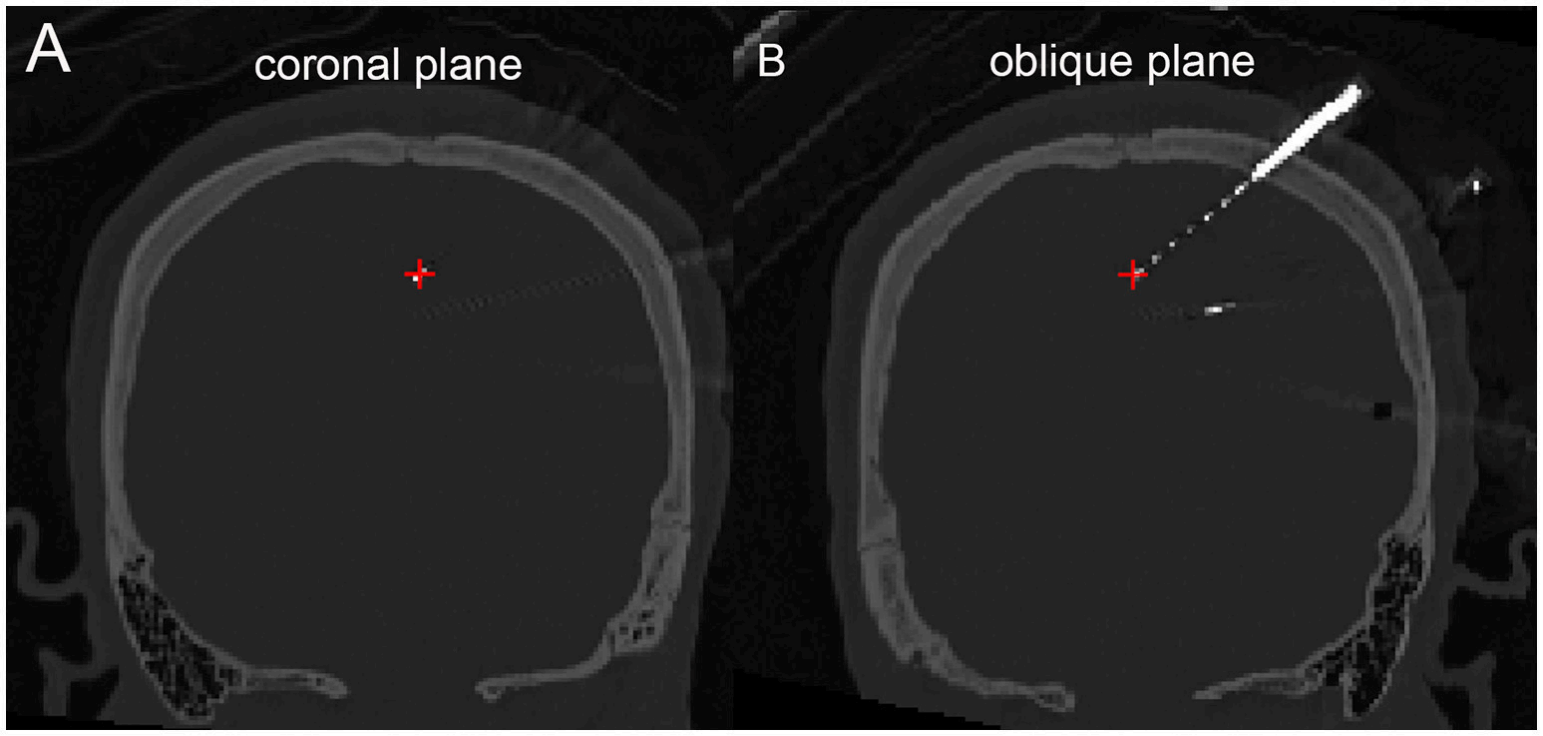
**Peri-coronal view for displaying an electrode strip**. **(A)** Showing typically orthogonal SEEG electrodes in coronal view. **(B)** Showing a full SEEG electrode displaying in a non-orthogonal coronal view.

The density map can be used to display many indices, including the group-level electrode distributions and neural activity distribution. An example was shown in volume and surface space using different smoothing parameters (Figure 4). The standard view of the rendered surface can be pictured in a full range of perspectives and superimposed with a predefined density map. The oblique view of the whole electrodes was useful to highlight a single track and check the crossing anatomy along it. Figure 5 showed a full SEEG electrode in peri-coronal view.

### Segmentation of iEEG electrodes

#### ECoG

**Figure 6:**
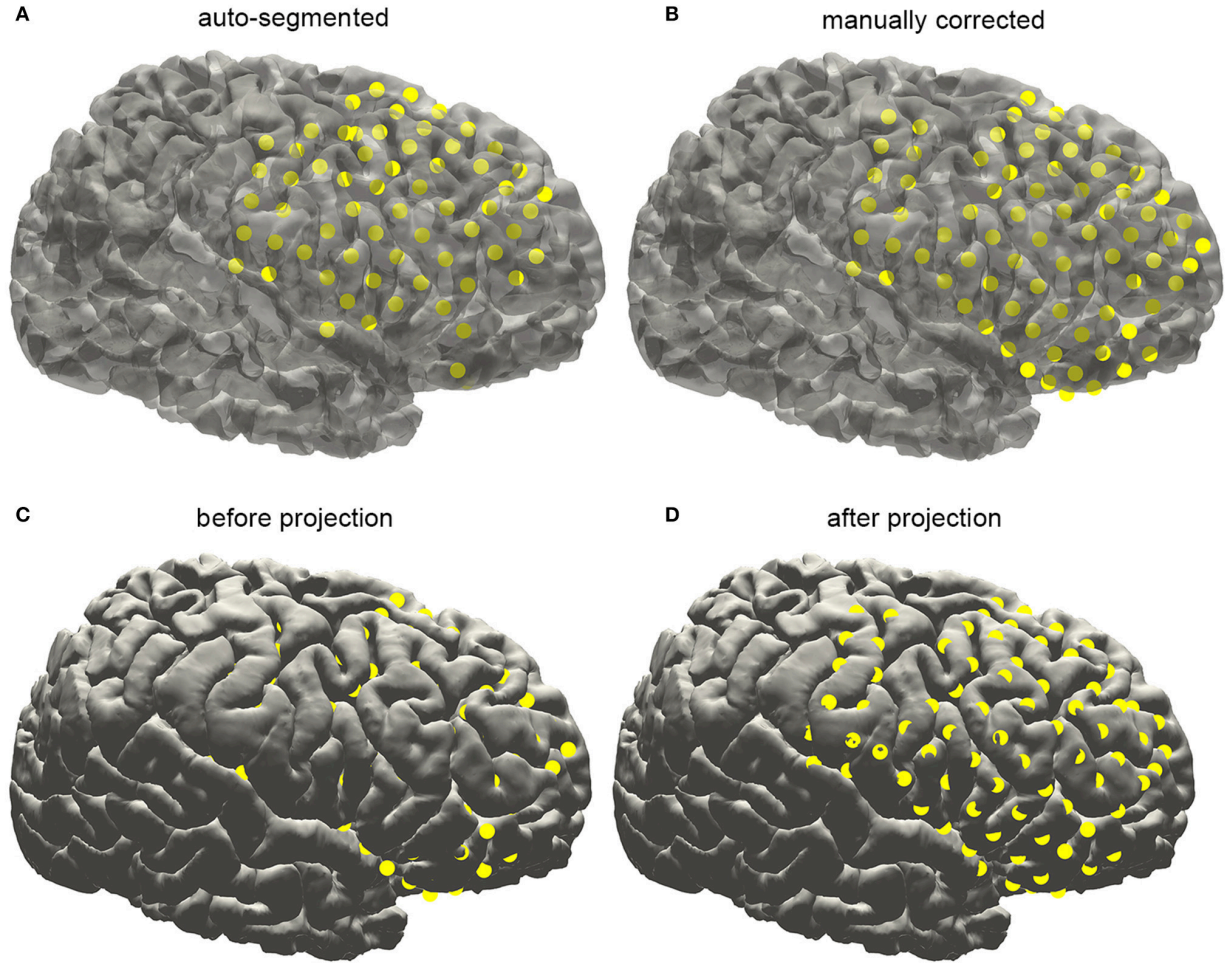
**Subdural electrode reconstruction, manual correction, and surface projection**. **(A)** The subdural grid contacts are detected by the automatic process. The missing and dislocated contacts can be easily recognized. **(B)** The final electrode arrays are displayed on the pial surface after manual correction. **(C)** Due to the brain deformation caused by electrode implantation, some electrode points appear to be buried beneath the cortical surface. **(D)** The electrode arrays are projected onto the brain surface.

With an appropriate brain mask, the ECoG auto-segmentation process was able to locate about 80–90% of the electrode contacts (Figure 6A). The manual corrections include removing some contacts wrongly detected and adding lost contacts. Electrode contacts identified can be compared before and after correction, which was shown in the Figure 6B for a typical patient.

**Figure 7:**
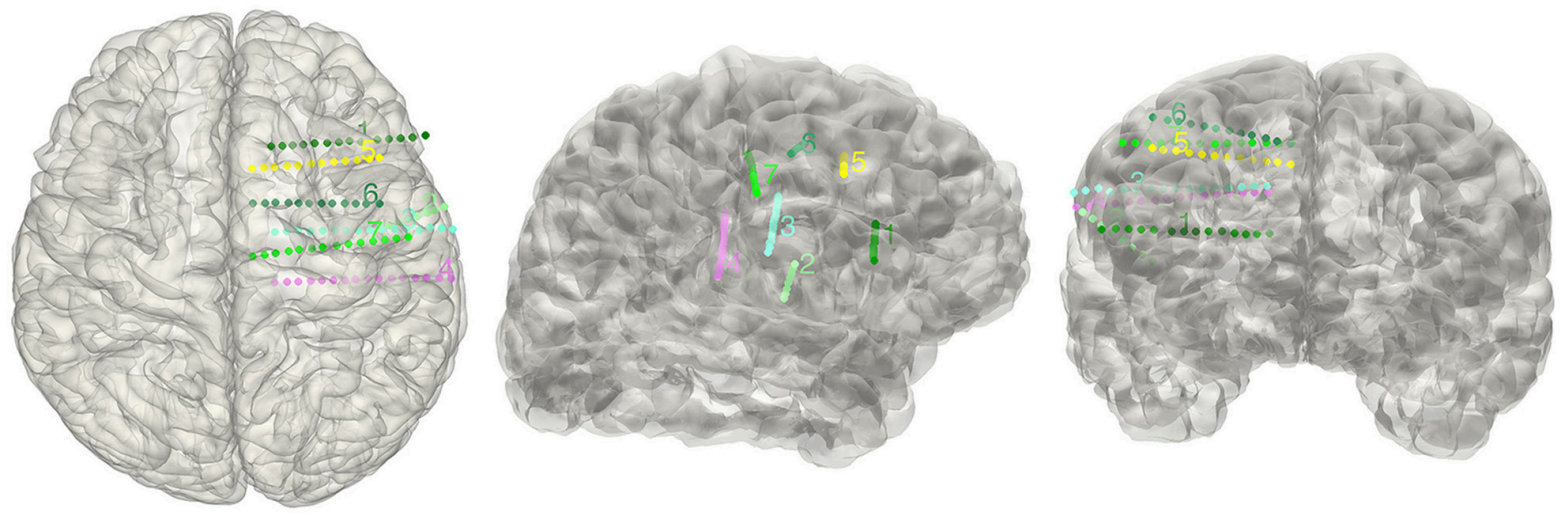
**Display electrodes of one patient**. Showing all the electrodes' relative positions on a rendered cortical surface using color and number to differentiate.

Electrodes implantation may induce structural deformation due to brain edema. The extent of displacement was associated with many factors such as the size of the implanted grid and the surgical operation. As shown in Figure 7C, many auto-segmented contacts appeared to be buried in the gray matter due to the tissue deformation. Hence a correction procedure was applied by projecting the segmented contacts first to a smoothed pial surface and subsequently back to the raw pial surface. Here we employed a minimal energy projection method to correct this deformation (Dykstra et al., 2012), which has been demonstrated to present a good reliability. The final segmentation process will output the estimated coordinate of each contact.

#### SEEG

The electrode segmentation provided a high accuracy estimation of the real electrodes positions. All of the contacts are color-coded and represented in relative positions in the transparent surface view (Figure 7). Similarly, this procedure output all the estimated contact coordinates.

### Anatomical localization and atlas-based parcellation

**Figure 8:**
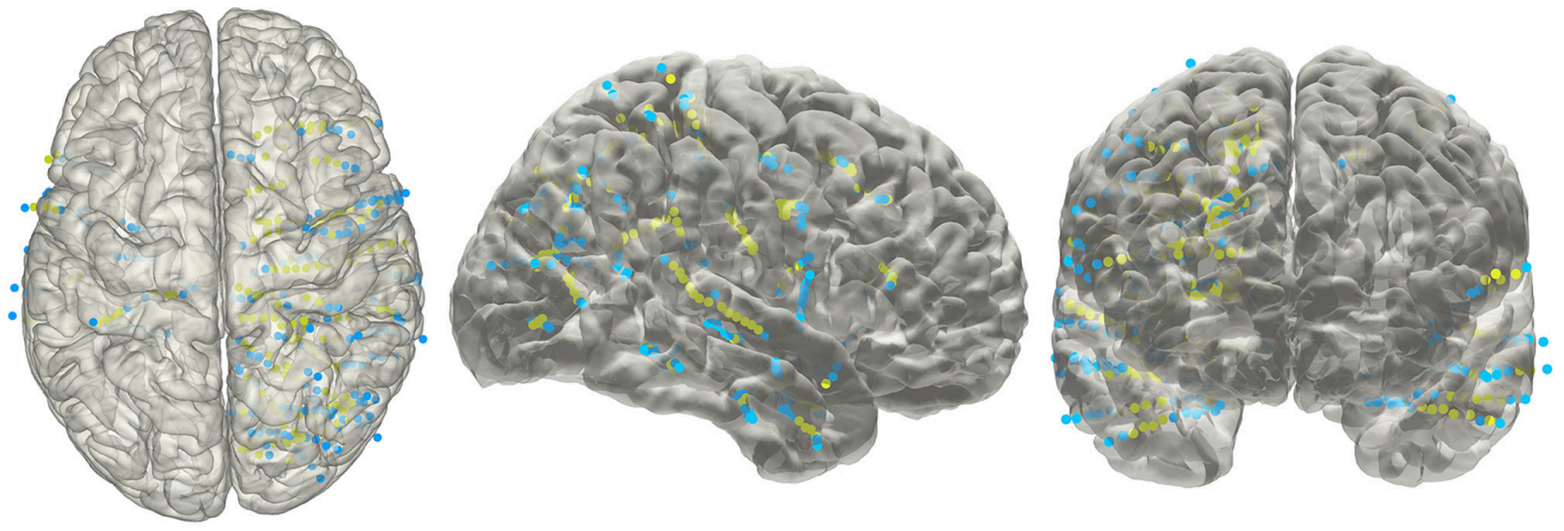
**Electrodes representation in standard space**. The electrodes' distributions of patients included in the validation process on a standard MNI152 surface, green dots indicate contacts in white matter area; blue dots indicate contacts in gray matter area.

The electrode information was regularly summarized into a text file, together with the probability value being to an anatomical area or a functional network. The electrode coordinates in the MNI152 space (Figure 8) were also exported for group statistics. The contents of the generated text file can be customized as well.

### Validation of SEEG electrodes reconstruction

Given that the subdural ECoG electrodes can be manually corrected in the segmentation procedure and the projection method has been approved to be effective (Dykstra et al., 2012), we didn't provide further validation for ECoG electrodes segmentation in this study. For with SEEG electrodes, validation procedure was performed in four patients. We chose a total of 20 contacts (4,7,4,5 for each patient) among all patients, which was visibly located on the brain surface; first we measured the relative Euclidean distance between each pair of contacts both on the intraoperative picture (which we consider as a plane) and the reconstructed surface for each patient, then we calculated the correlation between the two distances and use this correlation coefficient as the measure of consistency between true electrode locations and our reconstructed locations. For each patient, the correlation of the chosen distance pair was 0.99, 0.97, 0.98, or 0.98 respectively; thereby one can be confidential about the accuracy of our reconstruction process.

## Discussion

The accurate localization and parcellation of intracranial electrodes are crucial issues for medical diagnosis and scientific research. Several techniques are available for the localization of subdural or depth electrodes. In this study we proposed an integrated framework for anatomical and functional localization of intracranial electrodes by developing semi-automatic segmentation methods for ECoG and SEEG electrodes. In this study, the segmentation methods used commonly available preoperative T1 and post-operative CT as source images. After coregistering the CT with T1, we were able to locate and parcellate intracranial electrodes with minimal manual interventions. To achieve more accurate localization of the implanted electrodes, our methods utilized the advantages that have been proposed in previous studies including the center of mass calibration (Arnulfo et al., 2015) and surface projection (Dykstra et al., 2012). The cubic line-fitting algorithm was the first time introduced to solve the common problem of bending electrodes occurring for depth electrodes. Moreover, the estimation error of the localized electrodes in SEEG was less than or comparable with previous methods using a similar procedure (Hermes et al., 2010).

Usually tracking the locations of intracranial electrodes was time consuming and labeling each contact to a cortical region with high precision was also difficult. In this study we completed and integrated many useful functions in a single toolbox. Notably, this tool automated the whole processes mentioned above as much as possible to save human resources. Transformation of native space to the MNI standard space and the following cortical label provided us a convenient way to combine multiple patients and study the potential relationship between electrophysiology- and fMRI-based functional brain networks.

There were also several limitations in this study. First, sometimes it was difficult to automatically deal with close electrodes in SEEG and correctly seed all contacts in ECoG. Thus, the electrodes localizations proposed in the current method still required human interventions in manipulating the parameters, selecting the proper clusters and correcting the tracked electrodes. Second, the validation steps used in this study only measured the deviation of the cross point between electrode trajectories and the cortical surface in SEEG. Given that the inside-brain contacts cannot be pictured, we cannot quantitatively assess the accuracy of the reconstruction for the full depth electrode. Thus, the results obtained from 20 points may not implicate that the accuracy of the current method was superior to other published methods (Pieters et al., 2013).

In conclusion, we developed a toolbox for an effective localization and cortical label of intracranial electrodes with user interfaces as well as visualization utilities based on preoperative MRI and post-operative CT. To our best knowledge, this toolbox for the first time integrated SEEG and ECoG electrode localization and labeling processes and would provide a more precise and intuitive view for clinical assessment and human intracranial electrophysiological study.

## Ethics statement

This study was carried out in accordance with the recommendations of the Institutional Review Board of the Institute of Psychology, Chinese Academy of Sciences with written informed consent from all subjects. All subjects gave written informed consent. The protocol was approved by the Institutional Review Board of the Institute of Psychology.

## Author contributions

CQ and LW design the software, CQ, ZT, and LW develop the software, CQ, ZT, and LW write the manuscript, LR and WZ provided the experimental data. YP, YL and Lin W test this software. CQ and ZT contribute equally to this work.

### Conflict of interest statement

The authors declare that the research was conducted in the absence of any commercial or financial relationships that could be construed as a potential conflict of interest.
